# Mating Behavior of Manatees Documented in Mississippi Estuarine Waters

**DOI:** 10.1002/ece3.72831

**Published:** 2025-12-23

**Authors:** Holley Muraco, Matthew Virden, Megan Chevis, Keith Chenier, Maya Stratman, Amanda Free, Eric Sparks

**Affiliations:** ^1^ Coastal Research and Extension Center Mississippi State University Biloxi Mississippi USA; ^2^ Department of Animal and Dairy Sciences Mississippi State University Mississippi State Mississippi USA; ^3^ Department of Wildlife, Fisheries, and Aquaculture Mississippi State University Mississippi State Mississippi USA; ^4^ Mississippi Sound Estuary Program Biloxi Mississippi USA; ^5^ Mississippi‐Alabama Sea Grant Consortium Ocean Springs Mississippi USA

## Abstract

The *Trichechus manatus latirostris* (Florida manatee) is known to seasonally range into the northern Gulf of Mexico, but evidence of mating behavior in Mississippi has not previously been documented. On 24 September 2024, the first known instance of active manatee mating behavior in Mississippi waters was observed using an uncrewed aerial system (UAS) at the Grand Bay National Estuarine Research Reserve. Behavioral analyses, including all‐occurrence and continuous sampling methods, revealed mounting interactions characteristic of manatee reproductive behavior. The focal group consisted of three individuals, with one identified female (manatee B) and presumed male (manatee A) engaging repeatedly in mounting behaviors. Manatee C, sex unknown, did not participate in mating behaviors during the observation period. These findings suggest that Mississippi coastal habitats may support not only seasonal foraging but also reproductive activity, indicating a potential range expansion and reproductive plasticity of the species. This observation underscores the need for enhanced monitoring and conservation efforts in northern Gulf habitats to account for emerging manatee behaviors and potential establishment of reproductive areas beyond Florida.

## Graphical abstract

This study documents the first recorded instance of mating behavior by Florida manatees ( *Trichechus manatus latirostris* ) in Mississippi estuarine waters, observed opportunistically via a drone during a routine survey at the Grand Bay National Estuarine Research Reserve. The behaviors, including repeated mounting interactions, suggest that Mississippi coastal habitats may support not only foraging but also reproductive activity, with important implications for regional conservation and management strategies.

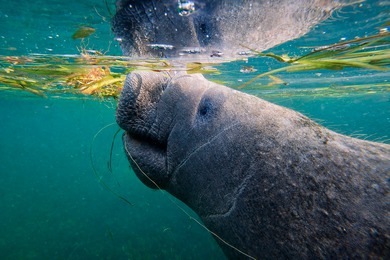

## 1 Introduction

**FIGURE 1:**
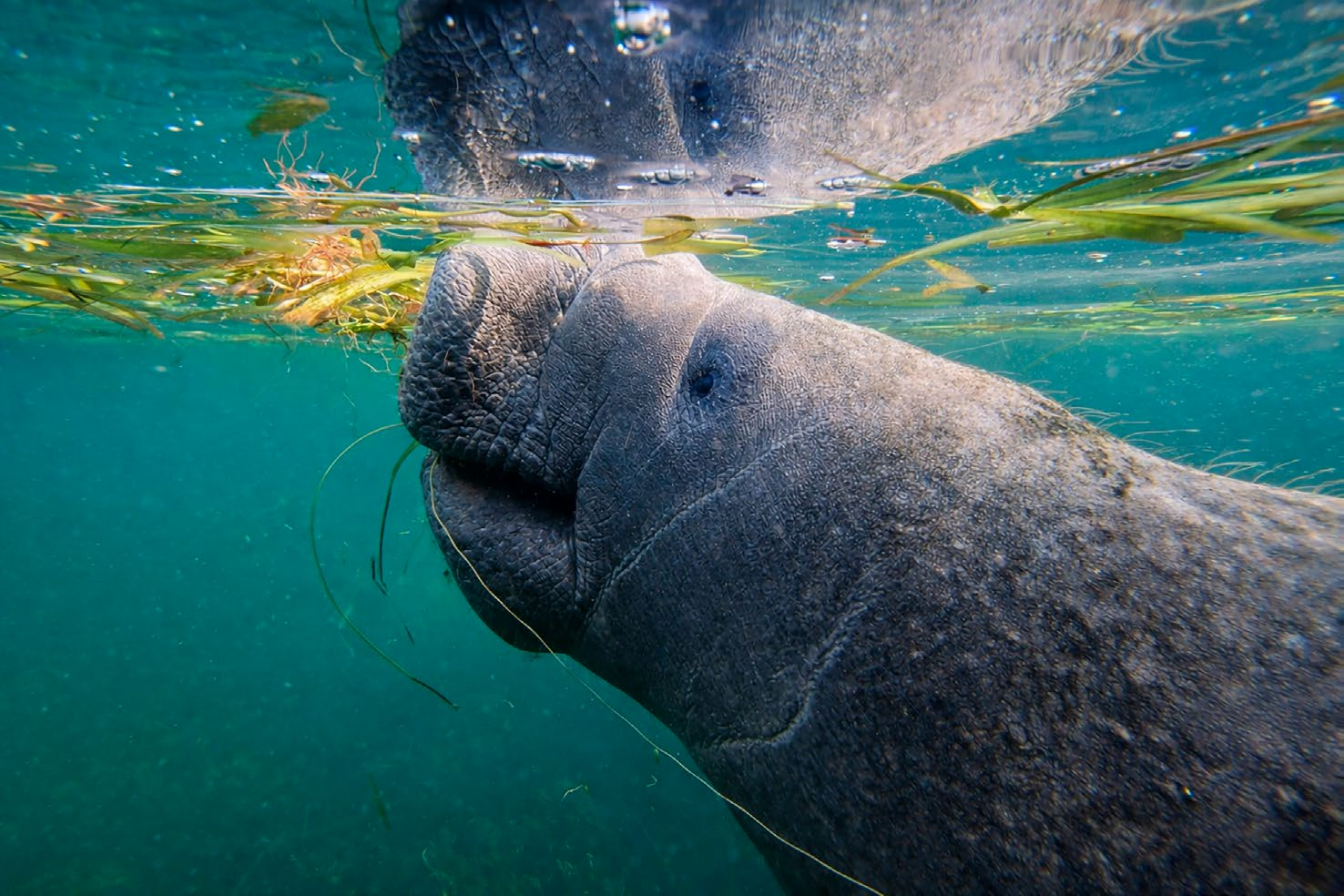
*Trichechus manatus latirostris* (Florida manatee).

There have been increasing sightings of *Trichechus manatus* (West Indian manatee), particularly *Trichechus manatus latirostris* (Florida manatee subspecies) in the northern Gulf of Mexico (nGOM), a region historically considered outside its typical range (Powell and Rathbun 1984; Fertl et al. 2005; Cummings et al. 2014; Aven et al. 2016; Hieb et al. 2017). Aven et al. (2016) tracked 13 manatees using satellite telemetry and photo‐ID from Mobile Bay, AL, across a region spanning from Tampa Bay, FL, to Lake Pontchartrain, LA. The study found consistent seasonal manatee migration patterns and high site fidelity in northern zones, especially in warmer months. Hieb et al. (2017) used over 1700 opportunistic manatee sightings collected via the Dauphin Island Sea Lab's Manatee Sighting Network (DISL/MSN) to analyze spatial and temporal trends in Alabama and Mississippi. Their results supported earlier findings (Aven et al. 2016), showing peak sightings during summer and early fall with increasing mortalities over the winter months due to cold stress (Figure 1).

Manatee social structure is generally characterized by fission–fusion dynamics, with transient groupings forming in response to ecological factors such as food availability, shelter, and mating opportunities (Rathbun et al. 1995; Preen 2004). Mating behavior in Florida manatees often involves the formation of “mating herds,” in which multiple males vigorously pursue and attempt to mate with a single estrous female over extended periods, sometimes spanning weeks and covering significant distances (Hartman 1979; Bengtson 1981; Rathbun et al. 1995). These interactions, while crucial to reproductive success, are energetically costly and can involve both adult and subadult males, highlighting a complex mating system shaped by sperm competition and female choice (Reynolds et al. 2004).

Florida manatees are known to breed year‐round but have seasonal peaks in reproductive activity, particularly during warmer months (Hartman 1979; Odell et al. 1995). Mating herds are normally observed between March and August with some sightings as late as October (Irvine 1983; Runge et al. 2017). While most reproductive studies focus on peninsular Florida, data from the nGOM show similar seasonality. Mating herds have been documented in Alabama waters in late summer (July–September), supporting local breeding behavior (Hieb et al. 2017). Sightings of mother‐calf pairs in Alabama and Mississippi have also been concentrated in June through September, consistent with movement into northern estuarine habitats during warmer months (Hieb et al. 2017). Warmer water temperatures (> 20°C) appear to facilitate more frequent breeding interactions (Deutsch et al. 2003).

Despite detailed behavioral descriptions in the wild, there remains a gap in understanding the physiological and endocrine correlations of manatee reproductive behavior. Early studies noted potential signs of estrus, such as vulvar swelling, and described mounting and inverted postures as potential behavioral indicators of reproductive state (Hartman 1979; Marmontel et al. 1992). Studies of captive female manatees have provided the opportunity to correlate specific behaviors with endocrine data. Observations have identified behaviors such as blowing bubbles, adopting inverted postures, and mounting as potential indicators of estrous cycles (Randall et al. 1999; Larkin 2000). Variations in reproductive hormone profiles suggest that while Florida manatees are considered to have year‐round reproductive potential, seasonal peaks occur, reflecting a flexible breeding strategy (Rathbun et al. 1995; Larkin 2000).

This study utilized opportunistic observations and video recordings of manatees engaging in mating activities in Mississippi waters during a nonrelated field activity using a small uncrewed aerial system (UAS). UAS's offer unique advantages for behavioral research and quantifying behavioral patterns (Christiansen et al. 2016; Torres et al. 2018).

## 2 Methods

### 2.1 Field Observation

**FIGURE 2:**
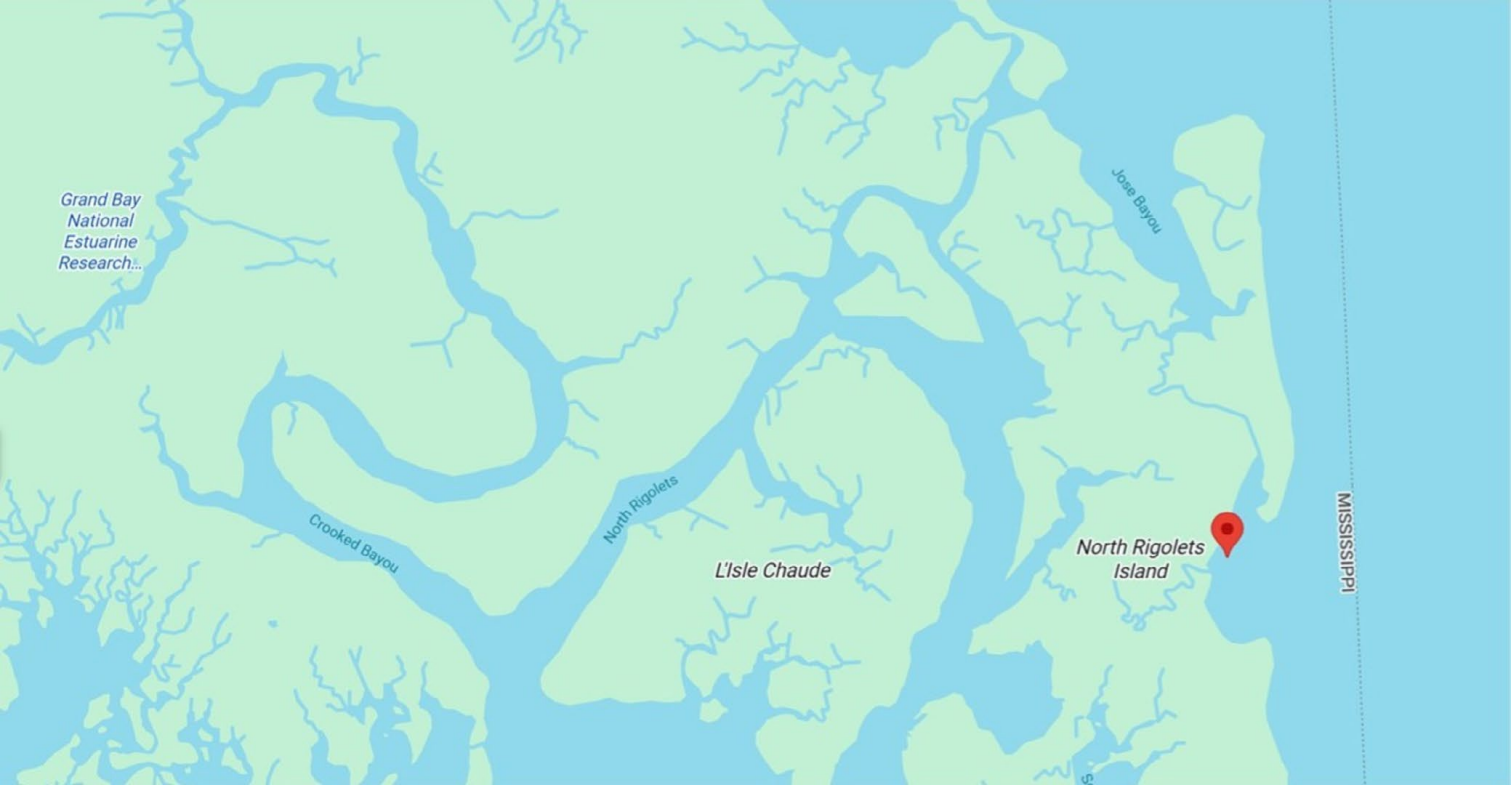
Map showing the location (red pin) of the observation at the Grand Bay National Estuarine Research Reserve in Jackson County, Mississippi.

On September 24, 2024 at 1200 CST, a team of UAS Federal Aviation Administration (FAA) part 107 licensed pilots were conducting preliminary assessments to test the accuracy of digital elevation models over salt marshes in Class G airspace using a 595‐g quadcopter UAS (DJI Mavic Air 2 s, www.dji.com) in the Grand Bay National Estuarine Research Reserve (GNDNERR) off North Rigolets Island (30.361728, −88.398746) (Figure 2). The GNDNERR encompasses approximately 18,400 acres of coastal ecosystems along the Mississippi‐Alabama border (NOAA 2024). Environmental and water quality conditions during the observation period included an air temperature of 29.1°C, wind speed of 3 MPH, water temperature of 30.1°C, salinity of 22.1 ppt., depth of 1.1 m, dissolved oxygen of 37.7, turbidity of 8, and pH 7.^1^

Manatees were identified via the UAS at 30.361676, −88.398321 (Figure 2), and were engaged in surface‐based behaviors. The pilot followed the guidelines of the Marine Mammal Protection Act, which prohibits harming or harassing a marine mammal. The UAS was maintained at a consistent altitude of 20 m upon the initial observation of the manatees. The manatees did not discontinue the behaviors, which would indicate level B harassment. Because the manatees did not stop the behaviors and did not swim away from the area, two concurrent videos were recorded, resulting in 7 min and 11 s of footage. The manatees were still engaged in the behaviors when the UAS was flown away from the location.

### 2.2 Video Analysis

**FIGURE 3:**
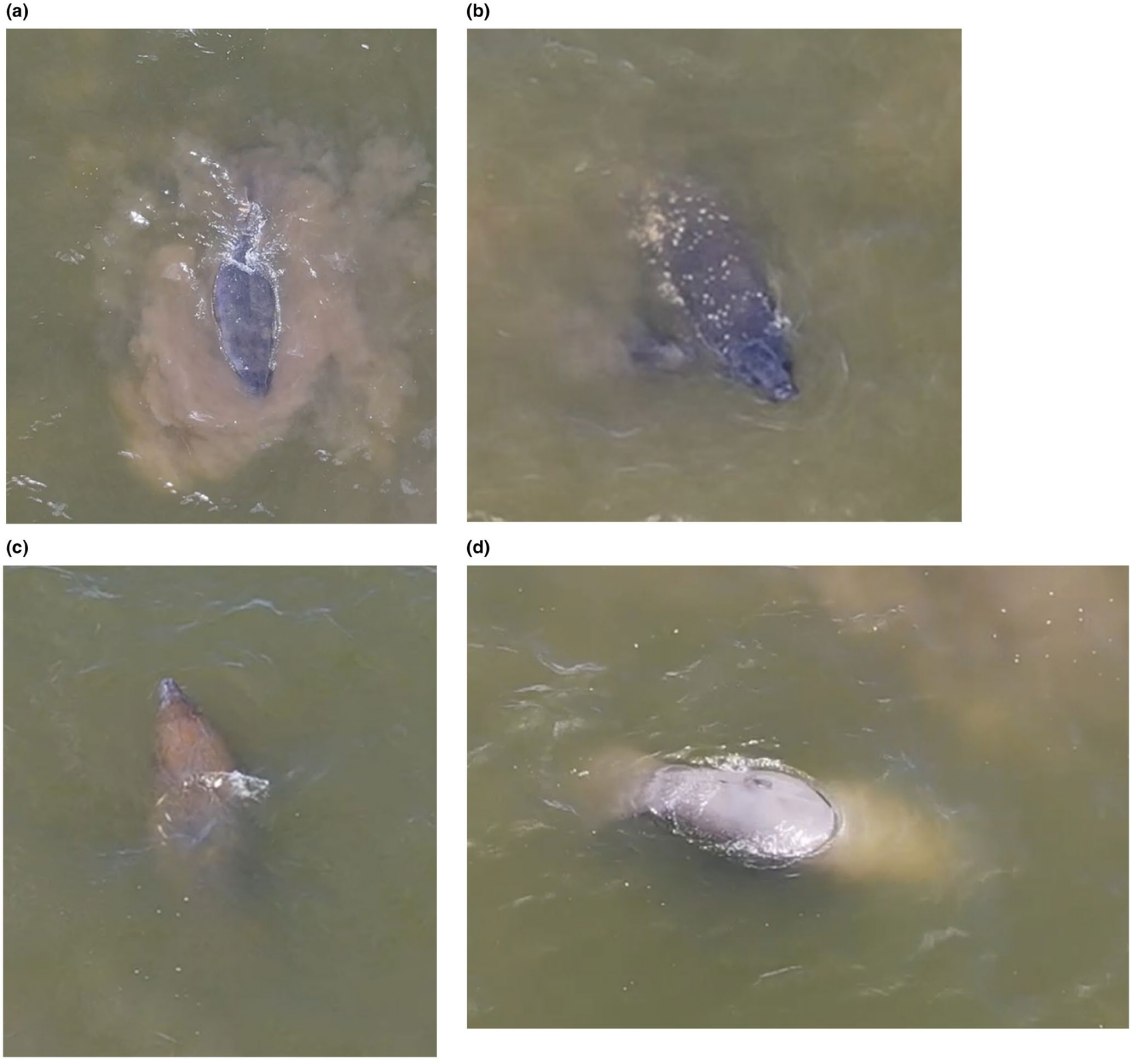
(a) Manatee A. Primary identifying mark is a boat scar on the right side at the junction of the body and paddle with a uniform gray coloration across the back. (b) Manatee B. Primary identifying mark consists of a white speckled pattern across the back and paddle. (c) Manatee C. Primary identifying mark of a boat scar along the left side of the back and a uniform brown coloration across the back. (d) Manatee B in an inverted swim position showing female genitalia above the water's surface.

The video footage was analyzed to identify the number of manatees present, defining marks that could be used for identification and to construct an ethogram of the observed behaviors. Three individual manatees with unique scarring were identified. The photos were shared with the Florida Fish and Wildlife Conservation Commission manatee photo identification program and the Dauphin Island Sea Lab Alabama manatee photo ID program (Figure 3a–c). These three manatees were not matched to any manatees in the existing photo ID catalogs (K. Rood, 2025 and R. Carmichael, 2024, personal communication). The manatees were easily observed when they were at or above the surface of the water despite the low visibility of the water.

### 2.3 Behavioral Ethogram

The manatees engaged in mating behaviors including frequent mounting. To determine if these behaviors were consistent with manatee mating behaviors traditionally observed in Florida waters, an ethogram was developed (Table 1). Horikoshi‐Beckett and Schulte (2006) developed an ethogram for captive female Florida manatees at Homosassa Springs Wildlife State Park that included 32 distinct behaviors and was used as a reference for this project.

**TABLE 1:** Ethogram of Manatee behaviors observed.

Behavior	Description
Mount	Abdomen of a manatee touches another manatee on the back, side or abdomen
Mounting sender	Mounts another manatee
Mounting receiver	Recipient of a mount from another manatee
Separate from mounting	Mounting manatees separate from one another so there is no contact but the manatees remain close in distance (< 100 cm)
Inverted Swim	Manatee turns upside down and swims
Swim Alone	Swim without any contact from another manatee in a distance > 100 cm
Not Visible	Underwater or out of view of the camera

### 2.4 Behavioral Analysis

Behavioral analyses were conducted using both all‐occurrence and continuous sampling methods (Table 2) to quantify manatee interactions using ZooMonitor software (Ross et al. 2016).

**TABLE 2:** Manatee all occurrence sampling results.

Behavior	Overall count	Manatee A	Manatee B	Manatee C
Mounting sender	21	15	6	0
Mounting receiver	20	5	15	0
Not visible	24	7	7	10
Separate	5	2	3	0
Swim alone	5	0	0	5
Inverted swimming	1	0	1	0

## 3 Results

### 3.1 Behavioral Sampling

The manatees observed had clear identification marks (Figure 3a–c) which allowed for the behavioral analysis of which manatee was the sender or the receiver of a mount (Table 2). The all‐occurrence sampling revealed that “Mounting Sender” behaviors were most frequently performed by manatee A (*n* = 15), while manatee B was most often the “Mounting Receiver” (*n* = 15). Manatee C primarily swam alone (*n* = 5) or was not visible (*n* = 10) and did not engage in mounting behavior during this observation. In total, mounting behaviors accounted for 21 sender and 20 receiver events across all individuals. The continuous sampling method demonstrated that “Mounting” occupied the greatest proportion of observed time, with a total duration of 237 s (56.4% of total observation time), followed by “Not Visible” at 160 s (38.1%) and “Not Mounting” at 23 s (5.5%). These behavioral observations suggest a clear reproductive context.

It is concluded that manatee A is likely a male based on the frequent mounting activity towards manatee B, a female based on genitalia present during an inverted swim position (Figure 3d). Manatee C's sex is unknown and did not engage in mounting attempts towards manatee A or B.

## 4 Discussion

This study was made possible through an opportunistic UAS operation looking at marsh vegetation in an estuary but ultimately led to a first observation of mating manatees in Mississippi waters. There is limited systematic data on breeding herd observations outside Florida, especially in Mississippi. Data are even further limited for mating efforts involving a single male and female manatee. These observations and behavioral data provide compelling new insights into manatee social and reproductive behavior in Mississippi coastal waters, specifically the first recorded evidence of mating behavior in the state. Historically, the Florida manatee is known to primarily breed within peninsular Florida, aggregating around warm water refugia during winter months and dispersing during warmer seasons (Hartman 1979; Rathbun et al. 1995).

Behavioral data collected using the UAS indicate that the manatees observed in this study were engaged in active reproductive courtship, consistent with “mating herd” dynamics described in Florida populations (Hartman 1979; Bengtson 1981). Manatee A was most often the mount sender, while manatee B was primarily the mount receiver, mirroring male‐dominant courtship roles documented elsewhere. Continuous sampling showed that “mounting” accounted for over half of the observed time, further supporting the interpretation of an intense reproductive interaction.

This observation has several ecological and conservation implications. The occurrence of mating related behaviors suggests that manatees visiting or residing seasonally in Mississippi are not merely transient but potentially establishing behaviors crucial for reproductive success. This supports hypotheses of increasing northward and westward expansion, possibly driven by warming coastal waters, reduced cold stress, and improved habitat conditions (Deutsch et al. 2003; Langtimm et al. 2011). The observation of mating behaviors implies that Mississippi estuarine and nearshore environments can provide adequate resources (e.g., freshwater inputs, forage availability, thermal refuge) necessary to support manatee reproduction. These conditions may position Mississippi's coastal habitats as future critical areas for the species. This discovery highlights the need for expanded monitoring and protection measures in Mississippi, especially given the potential for increased human‐manatee interactions (e.g., boat strikes, habitat degradation). Current management plans and educational outreach may need to be updated to reflect this new behavioral evidence. Observing mating behaviors outside traditional core ranges indicates possible gene flow with other Gulf of Mexico subgroups. This may have positive implications for genetic diversity but also complicates conservation strategies traditionally focused on Florida populations alone (Alvarez‐Alemán et al. 2010; Morales‐Vela et al. 2021).

Without the use of the UAS, this observation would not have been possible. The estuarine waters in this study have extensive and ever‐changing shallow areas that prevent boats from safely being able to navigate. Additionally, the waters are highly turbid with limited to no underwater visibility. More research is needed to better understand safety thresholds for flying UAS in the vicinity of manatees to ensure the animals are not harassed or harmed. Ramos et al. (2018) found that Antillean manatees ( *Trichechus manatus manatus* ) residing in clear waters exhibited strong avoidance responses to UAS flights when using a DJI Phantom II Vision early‐generation 1160‐g quadcopter piloted using rapid flight behavior and directional changes. Conversely, the UAS in this study, a 595‐g DJI Mavic Air 2s, maintained a steady altitude with no rapid flight behavior or directional changes over turbid waters. It cannot be concluded that the manatees in this observation did not detect the UAS; however, the manatees did not discontinue the mating behaviors for the duration of the flight. Flying a smaller, quieter UAS such as the DJI Mavic Air could be advantageous over larger, louder UAS when conducting manatee aerial surveys to reduce harassment potential.

The first documented mating behavior of manatees in Mississippi waters represents a significant expansion of our understanding of manatee ecology in the northern Gulf of Mexico. It underscores the importance of increased research and conservation investment in Mississippi's coastal ecosystems.

## Author Contributions

**Holley Muraco:** conceptualization (lead), data curation (lead), formal analysis (lead), funding acquisition (equal), investigation (lead), methodology (lead), project administration (lead), resources (lead), software (lead), supervision (lead), validation (lead), visualization (lead), writing – original draft (lead), writing – review and editing (lead). **Matthew Virden:** methodology (equal), writing – review and editing (equal). **Megan Chevis:** writing – review and editing (equal). **Keith Chenier:** writing – review and editing (equal). **Maya Stratman:** writing – review and editing (supporting). **Amanda Free:** writing – review and editing (equal). **Eric Sparks:** funding acquisition (lead), writing – review and editing (equal).

## Conflicts of Interest

The authors declare no conflicts of interest.

## Data Availability

All video recordings opportunistically recorded by the drone supporting the findings of this study are available at the following link: https://drive.google.com/file/d/1BjdU3hwKaOcphmhSIJTfIQKqQ9ACM8tS/view?usp=sharing.

## Endnotes

^1^
Data from Grand Bay National Estuarine Research Reserve Station GNDBHWQ.
